# Oxidative Stability of Stripped Soybean Oil during Accelerated Oxidation: Impact of Monoglyceride and Triglyceride—Structured Lipids Using DHA as sn-2 Acyl-Site Donors

**DOI:** 10.3390/foods8090407

**Published:** 2019-09-12

**Authors:** Qiang Wang, Yuejie Xie, Yuanyuan Li, Jianyin Miao, Hongbin Wu

**Affiliations:** 1 Innovation Center for Lipid Resource and Children’s Daily Chemicals, College of Biological and Chemical Engineering, Chongqing University of Education, Chongqing 400067, China; yjxie@sina.com (Y.X.); liyy@cque.edu.cn (Y.L.); 2 Guangdong Provincial Key Laboratory of Nutraceuticals and Functional Foods, College of Food Science, South China Agricultural University, Guangzhou 510642, China; miaojy8181@scau.edu.cn; 3 Institute of Agro-food Science and Technology, Xinjiang Academy of Agricultural and Reclamation Science, Shihezi 832000, China; wuhongbin1980@126.com

**Keywords:** oxidative stability, ω-3 polyunsaturated fatty acids, structured lipids, DHA, hydroperoxides

## Abstract

The current work aimed to clarify the effects of four structured lipids, including monoglycerides with docosahexaenoic acid (2D-MAG), diacylglycerols with caprylic acid (1,3C-DAG), triglyceride with caprylic acid at sn-1,3 and DHA at sn-2 position (1,3C-2D-TAG) and caprylic triglyceride on the oxidative stability of stripped soybean oil (SSO). The results revealed that compared to the blank group of SSO, the oxidation induction period of the sample with 2 wt% 2D-MAG and that with 1,3C-DAG were delayed by 2–3 days under accelerated oxidation conditions (50 °C), indicating that 2D-MAG and 1,3C-DAG prolonged the oxidation induction period of SSO. However, the inhibitory effect of α-tocopherol on SSO oxidation was reduced by 2D-MAG after addition of 2D-MAG to SSO containing α-tocopherol. 2D-MAG exhibited different antioxidative/pro-oxidative effects in the added/non-added antioxidants system. Compared to caprylic triglyceride, DHA at the sn-2 acyl site induced oxidation of structured lipids, thus further promoting the oxidation of SSO. The antioxidant was able to inhibit not only the oxidation of DHA in the SSO, but also the transesterification of sn-2 DHA to sn-1/sn-3 DHA in the structured lipid.

## 1. Introduction

Structured lipids (SLs) are a class of glycerides with specific molecular structure or function that chemically or enzymatically change the fatty acid (FA) composition or position distribution of the glycerol skeleton. The differences in structured lipids include not only the differences in the three FA species linked to the glycerol skeleton, but also the specific localization of FA on the glycerol skeleton (sn-1 and sn-3 positions outside the glycerol, or sn-2 sites in the middle). As fatty acids with special nutritional or physiological functions are attached to specific positions in the glycerol skeleton, structured lipids can utilize the advantage of the functions of various FAs, in addition to some or all properties of natural oils. As a new type of functional lipid, structured lipids contribute to digestion, energy absorption, timely supply of energy, and reduction of calories, and are important for nutrition and health care [1,2], because of which they are being widely utilized in the food [3], medicine [4], health products [5], and other industries.

The composition of FAs and their location on the glycerol carbon chain skeleton in SLs are different from those of natural materials. Some studies have showed that the type of acyl group on the glycerol skeleton of triglycerides determines the crystallization morphology of structured lipids, and these structured changes can effectively increase their solubility, reduce the specific calorific value of lipids, and improve their nutritional functions [5,6,7,8]. Currently, the academic community is intensively investigating the “grafting” of ω-3, ω-6 series unsaturated FAs to specific positions of the glycerol carbon chain skeleton, thereby improving the function of lipids and increasing their digestion and absorption rate in the body for the purpose of nutrition and health care. Consequently, owing to the high absorption rate of sn-2-site FA during lipid metabolism, Eicosapentaenoic Acid (*c*5*c*8*c*11*c*14*c*17C20:5, EPA), Conjugated Linoleic Acid (*c*-9,*t*-11C18:2, CLA), and α-Linolenic Acid (*c*9*c*12*c*15 C18:3, ALA) were used to synthesize structured lipids with sn-2-site polyunsaturated fatty acids (PUFAs), which not only improved the absorption rate of unsaturated FA, but also provided a substitute for low-calorie oil [9]. Robles et al. [10] showed that the absorption rate of lipids with palmitic acid at the sn-2 site was much higher than those with sn-1 or sn-3 site substitution, and the absorption rate of milk fat was significantly lower than that of breast milk, which was due to the presence of more sn-2-site palmitic acid in the fat of human breast milk. The differences in lipid absorption are mainly due to differences in the digestion and absorption mechanisms of lipids with varying structures. Studies have shown that the transesterification of some essential FA to the sn-2 position of glyceride can improve the absorption rate of the essential FA [11].

Long-chain ω-3 polyunsaturated fatty acids (e.g., DHA) play important roles in human health. Several studies have reported the health benefits of ω-3 FAs, particularly the advantages of DHA and EPA in cardiovascular, cerebrovascular, and postoperative recovery [12,13]. Owing to the presence of numerous unsaturated bonds, certain ω-3 FAs are easily oxidized and are unstable in the lipid system. Therefore, an important reason for the limited application of structured lipids with high content of ω-3 fatty acid was its high sensitivity to oxidative rancidity. Structured lipids with high ω-3 fatty acid are susceptible to oxidation and hydrolysis due to external conditions, leading to reduction in stability and freezing point, lack of physiological function, deterioration of food quality and nutrient content, and even food safety risk [14]. Frankel et al. [15] suggested that ω-3 fatty acids can be physically added to food for improving their nutritional value. However, the oxidation of oils is an essential limiting factor in this process. Therefore, methods of avoiding the loss of important FAs and improving the oxidation stability will be important for the storage and utilization of structured lipids.

Currently, there is no consensus regarding how structural isomerism or acyl group position such as sn-2 influenced oxidation stability of structured lipids. Previous studies have mainly compared the oil oxidation stability before and after esterification randomization [2,16,17]. The disadvantages of these studies are that the process of esterification randomization destroys natural antioxidants in the lipids and introduces various pro-oxidant artifacts, and the structured lipid cannot be directly compared with the crude oil. Studies on the oxidation stability of structured lipids with PUFA at sn-2 site in lipid systems were lacking. Therefore, it is necessary to investigate the oxidative stability of structured lipids using PUFA as sn-2 acyl-site donors in oil, which will contribute to minimizing the loss of important nutrients and the formation of potentially toxic reaction products (e.g., aldehydes and ketones) after the oxidation of PUFA structured lipids. To assess the oxidative stability and oxidation mechanism of PUFA structured lipids and to understand the differences in the oxidation of different PUFA structured lipids, the primary and secondary oxidation products of different structured lipids with/without DHA at sn-2 position in the stripped soybean oil (SSO) under accelerated oxidative storage conditions were studied. In addition, the oxidative stability and potential mechanism of oxidation of α-tocopherol in stripped soybean oil containing structured lipids with DHA under accelerated oxidation conditions were also investigated.

## 2. Experimental Procedures

### 2.1. Materials

2-Monoolein and diolein standards, propanal standards, and fatty acid methyl ester standards, were purchased from Sigma-Aldrich Chemical Co. (St. Louis, MO, USA). The immobilized lipase from *Rhizomucormiehei* (Lipozyme RM IM) was purchased from Novozymes A/S (Bagsvaerd, Denmark). Dry ethanol, *n*-hexane, acetone, chloroform, NaCl, caprylic acid, absolute ethanol, and acetonitrile were of HPLC or analytical grade and obtained from Fisher Scientific (Pittsburgh, PA, USA).

### 2.2. Stripped Soybean Oil Preparation

The method of stripped soybean oil was mainly adopted from Waraho et al. [18] with some modifications. All the glassware and sample bottles used in the experiment were soaked in 3 mM HCl solution overnight to remove the transition state ions, and then repeatedly immersed in double distilled water for 4 h for rinsing. Soybean oil was eluted with *n*-hexane to separate small polar components (e.g., tocopherols, free FAs, mono-, diacylglycerols, and phospholipids) that may interfere with the oxidative stability of the oil using a column of silicic acid and activated carbon. The 35 cm long chromatographic column with inner diameter of 3.0 cm was successively packed with 22.5 g silicic acid, 5.625 g activated carbon, and 22.5 g silicic acid, such that the packing material of the chromatographic column was divided into three layers. The chromatographic column was wrapped with aluminum foil to avoid oxidation of oil by light. Soybean oil (500 g) was dissolved in 500 mL *n*-hexane and then slowly poured into the column. The oil was eluted with 2 L *n*-hexane. To delay the oxidation of oil during the purification process, the stripped oil was collected in a container covered with aluminum foil and wrapped with ice. After washing, *n*-hexane was removed at 37 °C using a vacuum rotary evaporator (Heidolph, Schwabach, Germany). Residual traces of the solvent were removed by flushing nitrogen. Then, 300 g of the separated oil was separately transferred to several 3 mL vials, protected with nitrogen, and maintained at −80 °C for subsequent experiments. A small amount of the sample was taken and mixed with *n*-hexane, and spotted and developed on a thin-layer chromatography plate soaked with *n*-hexane:diethyl ether:formic acid (84:16:0.04, *v*/*v*/*v*) to confirm the removal effect of secondary components in SSO.

### 2.3. Preparation of 1,3C-2D-TAG Structured Lipids Via Two-Step Enzymatic Hydrolysis

#### 2.3.1. Synthesis of 2-Monoacyl Glycerol (2-MAG)

Structured lipid was synthesized according to Wang et al. [19]. 0.9 g algae oil and 3 g absolute alcohol were weighed and added to a 50 mL beaker. 0.4 g Lipozyme RM IM was added and magnetically stirred (IKA, Stauffen, Germany) at 40 °C for 5 h under a rotating speed of 200 rpm. After the reaction, the reaction mixture was centrifuged (Thermo Fisher Scientific Inc., Waltham, MA, USA) to remove the lipase. n-Hexane (30 mL) and 0.8 mol/L KOH (10 mL) were added to a certain amount of the centrifuged samples, and the solution was oscillated violently for 2 min, followed by 5 min of stationary incubation. Then, the lower alcohol solution was further extracted by adding 15 mL *n*-hexane, which was oscillated violently for 2 min and left undisturbed. After the formation of the two layers, the ethanol phase containing 2-MAGs was collected. The organic solvent was removed by rotary evaporation (40 °C), and the obtained samples were weighed and stored in a refrigerator at −20 °C for later use. The content of 2-MAG was quantified by comparing the peak area of 2-Monoolein standards.

#### 2.3.2. Synthesis of 1,3C-2D-TAG Structured Lipids

100 g MAG was weighed and 150 g caprylic acid, 25 g Lipozyme RM IM, 1000 mL *n*-hexane and 2 g 4A molecular sieve were added. The resulting mixture was magnetically stirred at 40 °C for 5 h under a rotating speed of 200 rpm. After the reaction was completed, the lipase was centrifuged at 5000 rpm for 10 min to obtain a final 1,3C-2D-TAG sample. To further purify the obtained 1,3C-2D-TAG structured lipids, the crude 1,3C-2D-TAG samples were loaded onto the chromatography column and eluted with *n*-hexane/diethyl ether (95/5, *v*/*v*) solution. After elution, the eluted fractions were analyzed using thin layer chromatography (TLC) and high performance liquid chromatography (HPLC). The purified 1,3C-2D-TAG structured lipids were collected according to the analysis results and stored at −20 °C for subsequent analysis and characterization.

#### 2.3.3. Analysis of Lipid Composition Using TLC

The lipid components (MAG, DAGs, TAGs, fatty acid ethyl esters) were analyzed using TLC. TLC plate was activated at 105 °C for 1 h before analysis. 10 μL samples were dissolved in 200 μL of *n*-hexane/diethyl ether (84/16, *v*/*v*), and 1 μL of each diluted sample was placed on the activated TLC plate, which was then placed in an oven at 120 °C to remove volatile solvents. Then a mixture of *n*-hexane:diethylether:formic acid (84/16/0.04, *v*/*v*/*v*) was spread onto the TLC plate containing the samples, and the fractions corresponding to different lipid classes were scraped from the TLC plates for further analysis.

### 2.4. Storage Methods and Oxidation Conditions of the Sample

**Figure 1:**
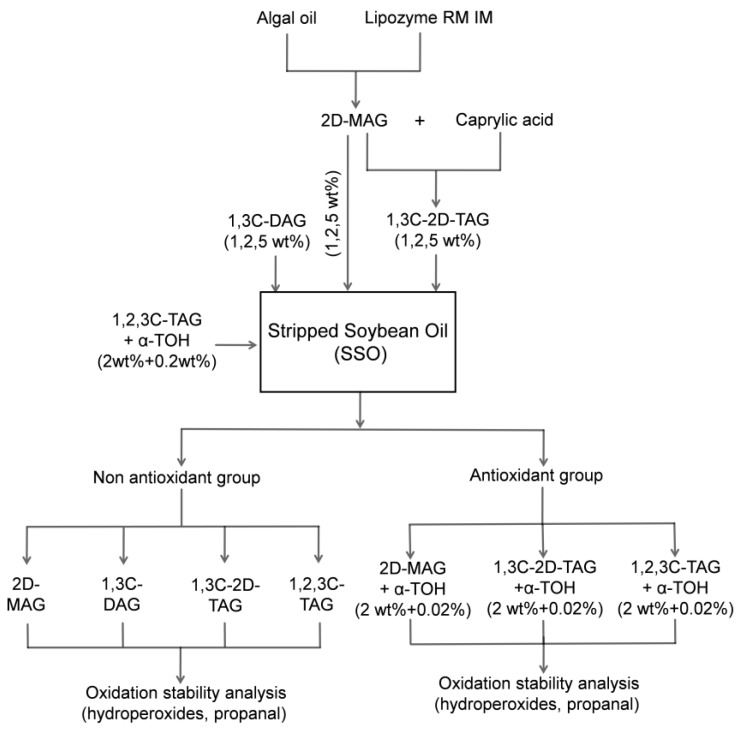
The experimental scheme for oxidative stability of stripped soybean oil with structured lipids.

The prepared MAG, DAG, TAG (1, 2, 5 wt%, respectively) and α-tocopherol (0.2 wt%) were added to the stripped soybean oil in different proportions as the Figure 1 experimental scheme. All of the samples were thoroughly mixed on the vortex oscillator to achieve uniform distribution. Then, 3 g of each group of samples was added to 3 mL glass vials and placed in oven at 50 ± 1 °C to avoid photooxidation for 20 days. The control group without additive was prepared at the same time. The primary oxidation products (hydroperoxide) and secondary oxidation products (propanal) were determined using the non-back sampling method at the same interval of oxidation time during storage.

### 2.5. Analysis of Structured Lipid Types Using HPLC

The MLM structured lipid product was subjected to HPLC analysis based on the method of More et al. [20] with modifications. Two microliters of the sample were injected into a HPLC (Inertsil ODS-2 column: 250 × 4.6 mm i.d., particle size 5 μm; Shimadzu, Kyoto, Japan) equipped with a UV detector (Shimadzu, Kyoto, Japan) set at 254 nm. The flow rate was maintained at 1 mL/min during the analysis. The mobile phase was acetonitrile: acetic acid (9/1, *v*/*v*). The composition of the mixture was determined based on the retention time and peak order, and the corresponding relative content was calculated from peak area.

### 2.6. Analysis of the Composition and Content of sn-2 Site FA Using GC-FID

The methyl esterification of fatty acid (FAME) was analyzed using the AOAC Official Method 996.01. The sample was saponified with 2 mL of 0.5 M NaOH-CH_3_OH at 60 °C for 30 min and then reacted with 14% boron trifluoride in a water bath at 65 °C for 5 min. After the reaction was completed, the FAME was extracted with about 2 mL *n*-hexane. FAMEs were characterized and quantified using GC-17A Shimadzu gas chromatograph equipped with AOC-5000 autosampler (Shimadzu, Kyoto, Japan) with 30 m × 0.32 mm column. The carrier gas flow rate of GC was 1.0 mL/min, and the split ratio was 100:0. The inlet and detector temperatures were fixed at 250 °C. The initial furnace temperature was held at 60 °C for 3 min, then increased to 175 °C at a rate of 5 °C/min and maintained for 15 min, and finally raised to 220 °C at a rate of 2 °C/min and held for 10 min. The FAs were qualitatively and quantitatively analyzed based on the peak time and relative peak area according to the FAME standards.

### 2.7. Determination of Peroxide Value (POV)

Lipid hydroperoxides were measured as the primary oxidation products using a method adapted from POV as a hydrogen peroxide content indicator used to reflect the oxidation stage according to the Shantha et al. [21] method with modification. Mixed solution containing isooctyl alcohol: 2-propanol (3:1, *v*/*v*) (1.5 mL) was added to 0.3 mL of sample and uniformly mixed in a vortex shaker (Benchmark Scientific, NJ, USA). The sample was centrifuged at 4000 r/min for 5 min, after which 0.2 mL of the upper organic phase was added to 2.8 mL of a mixed solution of methanol: 1-butanol (2:1, *v*/*v*), followed by the addition of 15 μL 3.94 M thiocyanate ammonium and 15 μL ferrous solution (obtained by mixing 0.132 M BaCl_2_ and 0.144 M FeSO_4_∙7H_2_O) and reacting in the dark for 20 min. Then, appropriate amount of the sample was used to measure the absorbance value at a wavelength of 510 nm using a spectrophotometer (ThermoSpectronic, Waltham, MA, USA).

### 2.8. Determination of Propanal

The propanal concentration was determined according to the method of Shantha et al. [21]. In brief, 1 mL sample was added to a 10 mL glass vial and the aluminum cap of the bottle was tightened. The measurement was performed after heating at 55 °C for 15 min in a gas chromatograph autosampler (Shimadzu, Kyoto, Japan) heating bath. After the solid phase microextraction (DVB/Carboxen/PDMS) fiber needle of the autosampler penetrated the stopper and absorbed the volatiles for 1 min, the SPME was transferred to the inlet and incubated for 3 min, followed by injection into the 65 °C GC column and incubation for 10 min. The inlet temperature was 250 °C and the split ratio was 1:5. The volatiles were separated on a Supleco 30 m × 0.32 mm Equity DB-1 column of 1 μm film thickness. The carrier gas was helium at 1.5 mL/min. The furnace temperature was set at 45 °C and held at that temperature for 5 min; next, the temperature was raised from 45 to 250 °C at a rate of 15.0 °C/min and maintained for 1 min. The FID detector (Shimadzu, Kyoto, Japan) was set to 250 °C. The standard curve was prepared with known concentration of propanal, and the concentration of propanal released from the sample was determined from the peak area. The experimental scheme for oxidative stability of stripped soybean oil with structured lipids is summarized in Figure 1.

### 2.9. Statistical Analysis

All tests were repeated thrice with freshly prepared samples. Data calculation was carried out using Origin 8.5. All of the data presented as means ± standard deviations (*n* = 3). The one-way ANOVA and Duncan’s multiple range tests were applied to determine significant differences between means (*p* < 0.05).

## 3. Results and Discussion

### 3.1. Purification of Soybean Oil Samples

The main components of commercial grade vegetable oils are glycerides, including monoglycerides, diacylglycerols, and triglycerides, which account for about 95% of the mass of commercial vegetable oils. Studies have shown that the concentrations of monoglycerides and diacylglycerols are much lower than those of triglycerides in animal fats and vegetable oils as the processing leads to partial hydrolysis of triglycerides to monoglycerides and diacylglycerols [18]. The remaining (5%) vegetable oil is mainly composed of unsaponifiable compounds (such as hydrocarbons, tocopherols, tocotrienols, phytosterols, chlorophyll, carotenoids, flavonoids, free fatty acids, polar polyphenols, and carbohydrates) and trace metal ions. These components are mainly derived from plant seed oil film or via hydrolysis during storage or application of pressure [22]. Although the amount is low, most of these components significantly affect the physical and chemical properties of oils, such as antioxidant (such as phenols) or pro-oxidation activities (such as free FA, metal ions, and chlorophyll) [23,24].

To reduce the effect of unsaponifiable oils on sample oxidation, the commercial vegetable oil was refined and stripped (removal of unsaponifiable matter). As shown in Table 1, the contents of stripped hydroperoxide and propanal were 2.22 mmol/kg and 9.83 μmol/kg, respectively, which were significantly lower than those in the commercial samples (*p* < 0.05). Khan et al. [25] showed that the stability of oil or water-in-oil emulsion prepared from stripped bean oil was significantly lower than that of the non-purified oil system, and the unsaponifiable matter in natural oil considerably affected the oxidation stability of oil. Therefore, the content of unsaponifiable matter in the stripped bean oils used in this study was low, and the oxidation interference effect of polar small molecules on the storage period of oils was also reduced, which ensured a more accurate and reasonable effect of different DHA sn-2 structured lipids on the oxidation of SSO.

### 3.2. Effect of Different Type of Structured Lipids on Oxidation Stability of SSO

Studies have shown that the concentration of diacylglycerols in vegetable oils is between 0.8 and 5.8% of the total oil content [26]. Therefore, to determine the effect of monoglyceride and diacylglycerol on the oxidative stability of SSO, 2 wt% monoglycerides with DHA (2D-MAG), diacylglycerols with caprylic acid (1,3C-DAG), triglyceride with caprylic acid at sn-1,3 and DHA at sn-2 position (1,3C-2D-TAG), and caprylic triglyceride (1,2,3C-TAG), respectively, were added into the SSO. Hydroperoxide and propanal were used as monitoring indicators to study the effects of different structured lipids on the oxidation of SSO under accelerated oxidation (50 °C).

**Figure 2:**
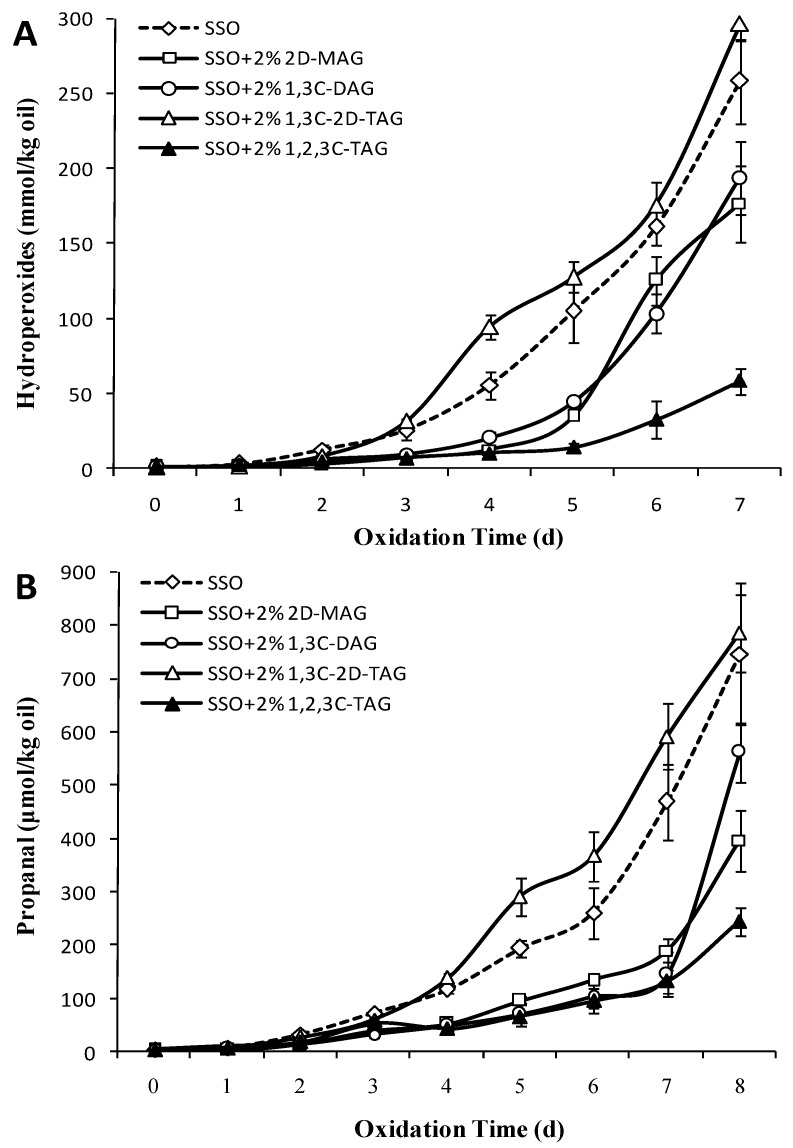
Effect of different structured lipids on the content of refined oil peroxide value (POV) (**A**) and propanal (**B**) in stripped soybean oil (SSO) (50 °C).

Hydroperoxides are the primary initial oxidation products of FAs. POV are commonly used to evaluate the oxidation state of FAs [27]. Figure 2A showed that after storage at 50 °C for 2 days, the POV of the 1,3C-2D-TAG group and the SSO blank group increased significantly compared to those of other experimental groups (*p* < 0.05), which reached 289.65 and 258.5 mmol/kg on the seventh day, respectively. Unlike the SSO of the blank group, the POV of 2D-MAG and 1,3C-DAG group began to increase significantly after the third day (*p* < 0.05), indicating that 2D-MAG and 1,3C-DAG prolonged the oxidation induction period of SSO to certain extent. Ohno et al. [28] also reported that the auto-oxidation of DAG at 50 °C was slower than that of TAG, and the oxidation induction period was longer than that of TAG. DAG and MAG are the main trace components in natural oils. DAG harbors a free hydroxyl group in the carbon chain, while MAG has two free hydroxyl groups. The differences in the physical and chemical properties of structured lipids may be due to the molecular structure of DAG, MAG, and TAG. Laszlo et al. [29] believed that the acyl migration rate of 1,3-DAG was slower than that of 2-MAG, as the presence of two FA groups in DAG led to a large deformation of the ring intermediate, thereby increasing the transition state energy barrier. All medium-chain saturated FAs were present at the 1,2,3C-TAG acyl sites, and therefore the POV of 1,2,3C-TAG began to increase slowly after 5 days of storage, eventually reaching 58.28 mmol/kg (7 days). This indicated that the oxidation of oil is related to not only the number of acyl groups attached to the carbon chain of glycerol, but also to the type of acyl FA, which considerably affected the degree and speed of oxidation of the oil. Wang et al. [30] showed that the oxidation rate of triacylglycerol was higher than that of diacylglycerol and monoacylglycerol. But our hypothesis is that the oxidation rate of structured lipid was correlated with the type of fatty acid attached to glycerol. Although the content of unsaturated FAs in DAG was higher, its oxidation rate was still significantly lower than that of TAG in our work. One possible speculation is that the transfer reactions of intramolecular free radical are faster than that of the intermolecular free radical.

The structure of FA was completely destroyed, and secondary oxidation products such as aldehydes, ketones, acids, and alcohols were further formed when a large amount of hydroperoxide accumulated during lipid oxidation. Among them, the change in propanal content was considered one of the most important indicators for evaluating the secondary oxidation products of oils. Figure 2B showed the effect of 2D-MAG, 1,3C-DAG, 1,3C-2D-TAG, and 1,2,3C-TAG on the secondary oxidation products of SSO. After storage for 3 days at 50 °C, the content of propanal in 1,3C-2D-TAG increased significantly (*p* < 0.05), and the rate of increase was significantly higher than those of the other groups. The content of propanal in the SSO without any added substance (SSO blank group) was 69.52 μmol/kg on the fourth day. Although there was a significant increase from the previous day, the content of propanal in the SSO blank group was lower than that of 1,3C-2D-TAG. Compared to that on the sixth day, the propanal content on the 7th day of the SSO group increased by 79.6%, indicating that there was an oxidation index period and that secondary oxidation products were formed in the SSO blank group on the sixth day. Among all experimental groups, the oxidation process of the 1,2,3C-TAG group was relatively slow, and the propanal content was only 153.08 μmol/kg on the eighth day. The propanal content of 2D-MAG and 1,3C-DAG increased significantly on the seventh day (*p* < 0.05), indicating that the oxidation induction period of 2D-MAG and 1,3C-DAG was delayed by 2–3 days compared to that of the 1,3C-2D-TAG and SSO blank group.

### 3.3. Effect of Different Concentrations of MAG on Oxidation Stability of SSO

**Figure 3:**
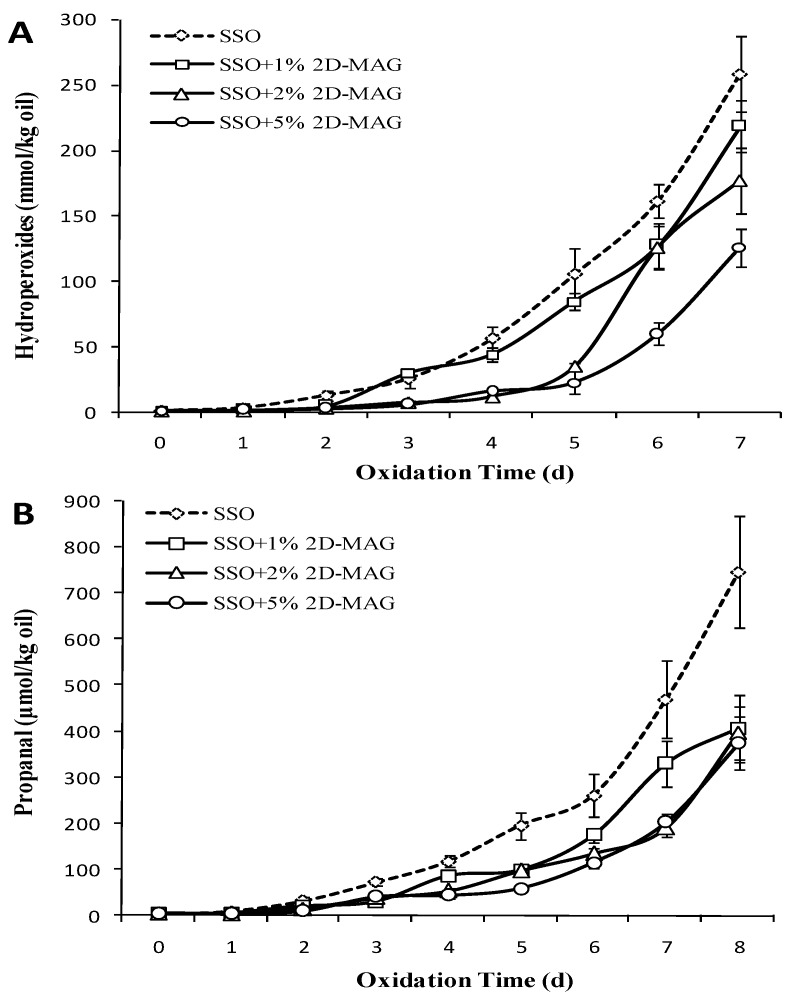
Effect of different concentrations of 2 wt% monoglycerides with DHA (2D-MAG) structured lipids on the content of refined oil POV (**A**) and propanal (**B**) in SSO (50 °C).

Figure 3A showed the effect of different concentrations of MAG on the POV of stripped soybean oil. The POV of the SSO control group began to increase slowly from the second day and reached the maximum value of 258.5 mmol/kg on the 7th day. Similar to SSO, 1% 2D-MAG had negligible effect on the POV of stripped soybean oil. However, the POV of the group with higher concentration (2%) of 2D-MAG was lower than that of SSO blank group. The group with the highest concentration (5%) of MAG showed significantly delayed oxidation of oil (*p* < 0.05), and the lag period formed by POV was extended to 5 days, which indicated that addition of more 2D-MAG (2–5 wt%) prolonged the oxidation induction period of oil to certain extent. Chen et al. [16] confirmed that addition of oleic acid monoglyceride (0.5%, 1.5%, and 2.5%) to stripped soybean oil inhibited the formation of SSO hydroperoxides to varying degrees. Our results of this study also revealed that addition of higher concentration of 2D-MAG was able to significantly slow down the SSO oxidation.

Studies have shown that there is an oxidation induction period of 1 day and 2 days for stripped soybean oil at 50 and 60 °C, respectively [31]. Figure 3B showed that the propanal level on the third day of each group was higher than that on the second day (*p* < 0.05). Among them, the propanal content (72.62 μmol/kg) on the third day of the SSO blank group was significantly higher than that of other groups. A large amount of propanal was formed in the SSO blank group, and it reached the highest value of 747.12 μmol/kg on the 8th day. Addition of different amounts of 2D-MAG inhibited the formation of SSO propanal, among which 5% 2D-MAG showed the most significant decrease in the amount of propanal, and the propanal produced by the oil at this ratio was the lowest on the 8th day (μmol/kg). Studies have also confirmed that 0.05–2.5% MAG had no effect on the production of non-stripped soybean oil POV and hexanal [18]. However, when MAG was added to SSO, the POV and hexanal tended to decrease. This indicated that in the presence of antioxidants (such as phenols), MAG was not the major factor affecting the oxidation process. This may be because the content of MAG in natural oils was small, and its ability to promote oxidation or inhibit oxidation cannot be completely realized. Thus, it can be deduced that the antioxidant capacity of 2D-MAG is more effective in the SSO system without trace amounts of polar substances, which protects SSO from oxidation.

### 3.4. Effect of Different Concentrations of 1,3C-DAG on the Oxidation Stability of SSO

**Figure 4:**
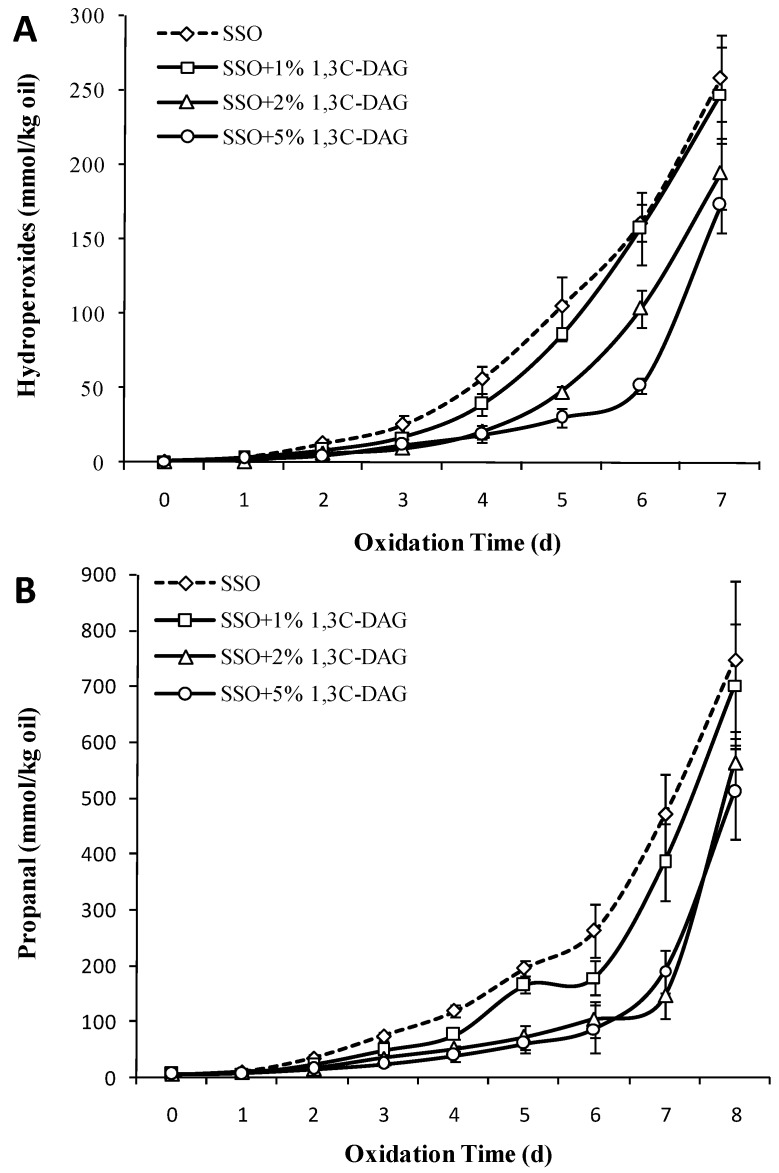
Effect of different concentrations of diacylglycerols with caprylic acid (1,3C-DAG) structured lipids on the content of POV (**A**) and propanal (**B**) in SSO (50 °C).

Diglyceride was a natural ingredient in many edible oils, and its content in most cases is below 5%. As glycerol has three positions at which acyl groups can be introduced, diacylglycerols are usually in the form of sn-1,3. Figure 4A showed that the SSO hydroperoxide content began to increase significantly from the fourth day after adding 1% 1,3C-DAG, and then was similar to that of the group without addition of 1,3C-DAG (*p* > 0.05). The POV of the group with higher concentration of 1,3C-DAG (2 wt%) began to increase significantly on the fifth day and was 194.33 mmol/kg on the seventh day. Hydroperoxide accumulated slowly in the group with 5% added 1,3C-DAG, which increased significantly on the sixth day, and reached 173.07 mmol/kg on the seventh day (increase by 235.1%). Studies [32] have shown that low doses of diacylglycerols can reduce the surface tension and increase the diffusion of oxygen in oils; however, high doses of diacylglycerols accumulate at the interface of oils to form a barrier and prevent further dissolution of oxygen. In addition, Qi et al. [33] also showed that pure DAG prepared from soybean oil has better oxidation stability than stripped soybean oil. 1,3C-DAG oil had a 22-day oxidation induction period, while soybean oil showed only 11 days of oxidative induction. Based on this, we believe that a low content of 1,3C-DAG might not change the oxidation pathway and induction period of SSO, while higher concentrations of 1,3C-DAG significantly inhibited peroxidation.

Figure 4B showed that as the concentration of 1,3C-DAG increases, the inhibiting effect of propanal in the SSO system becomes obvious. The propanal content after the addition of 1% of 1,3C-DAG began to increase significantly on the seventh day (*p* < 0.05), while those after the addition of higher concentrations of 1,3C-DAG did not. Chen et al. [16] showed that diacylglycerols (0.01–2.5%) prevented lipid oxidation in oil-in-water emulsions better than monoglycerides, and its ability to inhibit the production of hexanal in oils was also stronger. The oxidation resistance of diacylglycerols may be their ability to crosslink and form a physical barrier, which reduced the interaction or contact surface between unsaturated FAs and oxygen.

### 3.5. Effect of Different Concentrations of 1,3C-2D-TAG on the Oxidation Stability of SSO

**Figure 5:**
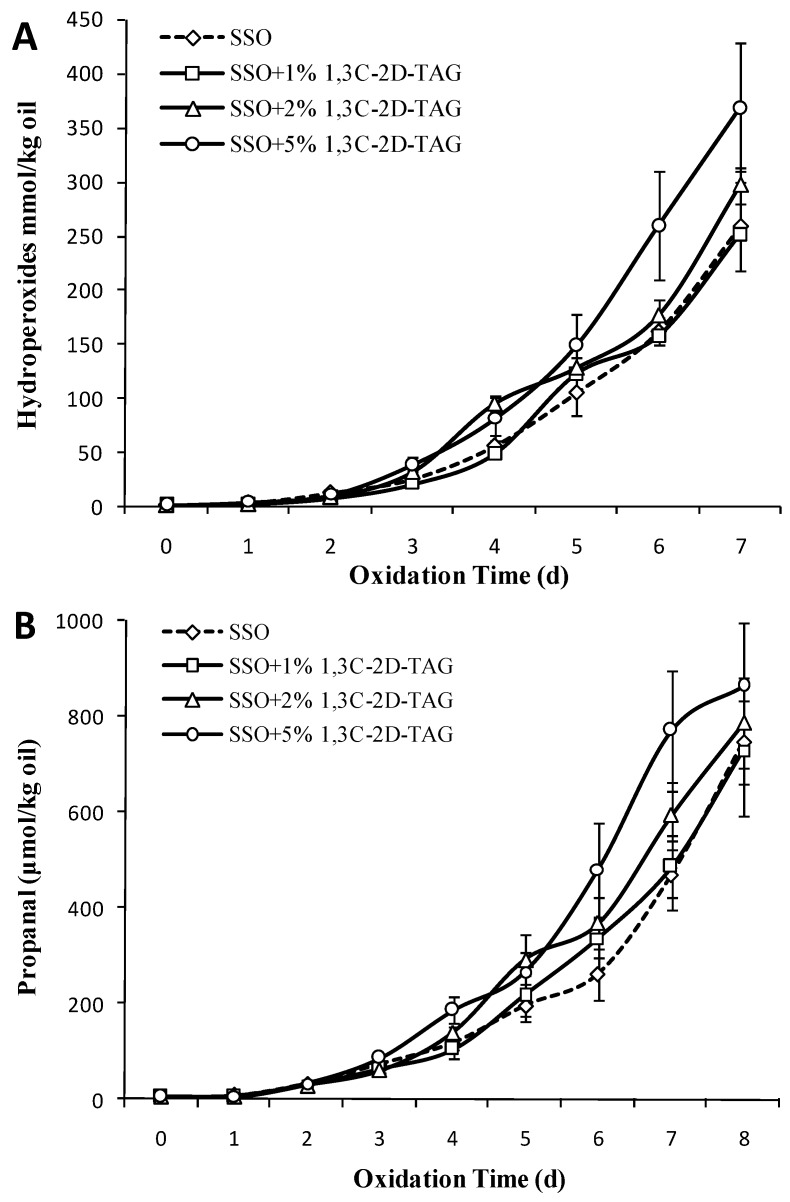
Effect of different concentrations of triglyceride with caprylic acid at sn-1,3 and DHA at sn-2 position (1,3C-2D-TAG) structured lipids on the content of POV (**A**) and propanal (**B**) in SSO (50 °C).

Raw soybean oil is highly susceptible to oxidation under accelerated conditions, owing to the presence of 7% α-linolenic acid and 50% linoleic acid. Figure 5A shows the effect of adding different amounts of 1,3C-2D-TAG on the oxidation of purified SSO. The POV began to increase on the third day with the addition of 1% 1,3C-2D-TAG. With increase in storage time, the change in POV content in the experimental group with 1% 1,3C-2D-TAG was basically identical to that in the SSO blank group (*p* > 0.05), indicating that addition of low doses of 1,3C-2D-TAG did not affect the oxidation process of SSO. However, the POV content of the group with high concentration of 1,3C-2D-TAG (5 wt%) was significantly higher than that of the SSO control group and the group with low concentration of 1,3C-2D-TAG (*p* < 0.05) from the third day, indicating that oil prepared with higher concentration of 1,3C-2D-TAG had lower oxidation stability. Rocha-Uribe et al. [34] demonstrated that the oxidative stability of lipids was related to CLA content and distribution of FA position, and the distribution of CLA at sn-2 sites was not conducive to the stability of the structured lipid. There are two main ways for degrading macromolecular FAs. One involves decarboxylation and decarbonylation, followed by C–C bond cleavage to produce hydrocarbon radicals or carbon ions; the other involves disruption of the hydrocarbons, followed by decarboxylation and decarbonylation to create short chain molecules. These two approaches compete with each other. Based on the DHA structure of the 1,3C-2D-TAG structured lipid at the sn-2 position, 1,3C-2D-TAG has partial polarity, and the probability of the exposure of unsaturated long chain of DHA in structured lipids is more. Therefore, the 1,3C-2D-TAG structured lipid is more likely to undergo decarboxylation and decarbonylation reactions after the bond cleavage of DHA hydrocarbons.

After initiation of lipid oxidation, the oxidation reaction turned into an autocatalytic process, which generated alkyl and hydroxyl radicals that continuously attacked and oxidized the unsaturated FAs. Figure 5B shows that the propanal content of the oil supplemented with 5% 1,3C-2D-TAG starts increasing rapidly on the fourth day, and reaches the maximum value of 860.2 μmol/kg on the eighth day. Compared to the SSO blank group, addition of low concentration of 1,3C-2D-TAG can also increase the formation amount of propanal to some extent, which indicated that the 1,3C-2D-TAG prepared in this study does not have commendable oxidation stability. Yang et al. [35] also confirmed that MLM-type structured lipids possessed low antioxidant capacity, which is possibly because the increase in transesterification during the preparation of structured lipids reduced the antioxidant capacity of raw lipids. In addition, the oxidation rate of free FAs in samples is higher than that of their esterification, which is also one of the reasons for the decrease in oxidation stability. Therefore, it is necessary to add appropriate antioxidants to protect the new structured lipids.

### 3.6. Effect of Different Concentrations of 1,2,3C-TAG on the Oxidation Stability of SSO

**Figure 6:**
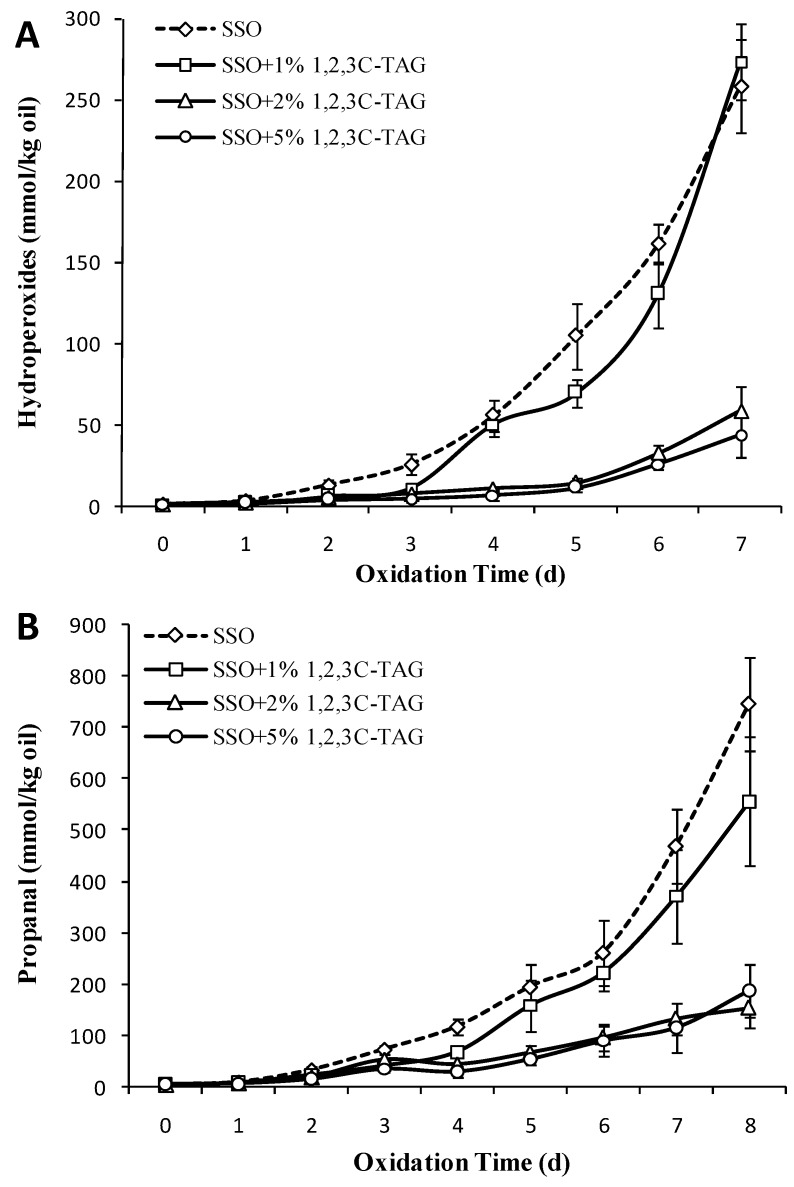
Effect of different concentrations of caprylic triglyceride (1,2,3C-TAG) structured lipids on the content of POV (**A**) and propanal (**B**) in SSO (50 °C).

Figure 6A showed that the effect of adding different amounts of 1,2,3C-TAG on oxidation inhibition of SSO vary considerably. Although the POV content of the sample with 1% 1,2,3C-TAG increased on the third day, the whole storage process had negligible effect on the change of SSO lipid (*p* > 0.05), except for the significant difference in POV content compared to the SSO blank group on the fifth day. With an increase in the amount of added 1,2,3C-TAG, the content of POV in the lipid began to increase slowly on the fifth day (*p* < 0.05), and the POV with 5% 1,2,3C-TAG was only 43.6 mmol/kg on the seventh day of storage. This indicated that the high concentration of 1,2,3C-TAG can delay the oxidation of the SSO, which may be related to the higher saturation of the medium chain caprylic acid in 1,2,3C-TAG lipids in this study.

Similar to the results of the POV content, the effect of different concentrations of 1,2,3C-TAG on propanal content during oxidation of the SSO system correlated with the amount of added 1,2,3C-TAG. As the amount of added 1,2,3C-TAG increased, the rate of increase in propanal content in lipids was significantly lower, which also indicated that addition of high concentrations of 1,2,3C-TAG was beneficial for delaying the oxidation induction time of SSO. Guillén et al. [36] observed that lipids with highly unsaturated FAs in triglycerides had the fastest oxidative degradation rate. Compared to that of the structured lipid 1,3C-2D-TAG, the oxidation effects of the same concentration of two kinds of TAG on the SSO lipid system was significantly different. When DHA was attached to the sn-2 acyl position, the oxidative stability in the triglyceride system decreased, indicating that the type of the FA at the sn-2 acyl position significantly affected the degree and speed of oxidation of the lipid. The order of oxidation rate of triglycerides may be caused by the faster occurrence of intramolecular free radical transfer reaction than intermolecular free radical transfer reaction. Therefore, it is speculated that triglycerides with more stable intramolecular structure has higher oxidation stability.

### 3.7. Effect of 2D-MAG on the Oxidation Stability of SSO with α-Tocopherol

**Figure 7:**
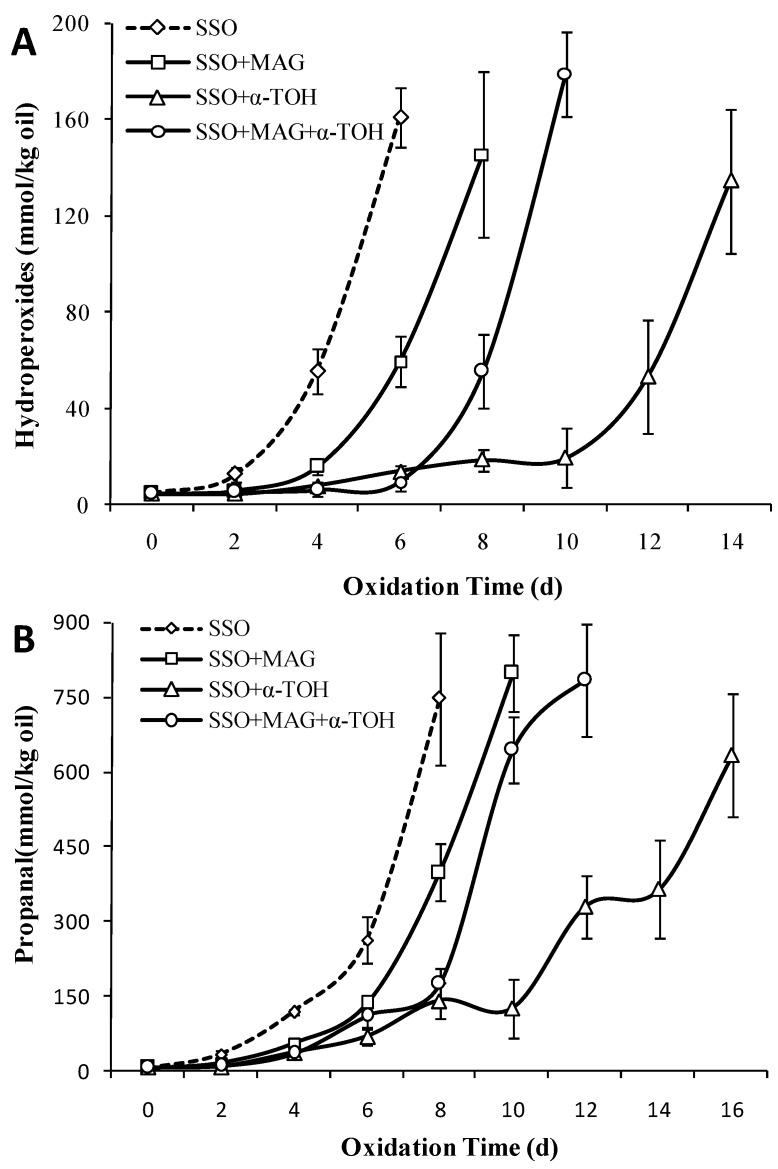
Effect of 2D-MAG structured lipids on the content of POV (**A**) and propanal (**B**) of stripped soybean oil with and without α-tocopherol (50 °C).

As shown in Figure 7A, the oxidative lag period of SSO was shorter, and the hydrogen peroxide value on the 4th day increased significantly to 55.8 mmol/kg (*p* < 0.05). The oxidative lag period of lipids with added 2D-MAG was significantly delayed for 2 days, indicating that 2D-MAG inhibited the oxidation of SSO to certain extent. The oxidation of SSO with 0.2 wt% α-tocopherol was inhibited (*p* < 0.05), and the oxidative lag period reached 10 days. When α-tocopherol was exhausted, SSO started undergoing oxidation on the twelfth day, and quickly reached 134.64 mmol/kg on the fourteenth day. After adding 2 wt% 2D-MAG to SSO with α-tocopherol, the oxidation of SSO was significantly inhibited during 0–6 days of storage, while the hydrogen peroxide value of SSO increased rapidly in the subsequent storage process, reaching 179.45 mmol/kg. As shown in Figure 7B, the oxidation of SSO in the control group started on the fourth day and the content of propanal increased rapidly, indicating that the hydroperoxide had been further degraded into secondary volatile oxidation products during SSO storage. After the addition of α-tocopherol, the oxidation of SSO was not significantly accelerated until the tenth day, and thrice the amount of propanal was produced on the 12th day, indicating that α-tocopherol inhibited not only the increase of SSO hydroperoxydes, but also the increase in propanal content.

It was noteworthy that compared to that of the SSO blank group without 2D-MAG, the addition of 2D-MAG to α-tocopherol-supplemented-SSO lipid shortened the oxidation induction period of the lipid by 4 days, indicating that the addition of 2D-MAG promoted SSO oxidation. However, the addition of 2 wt% 2D-MAG to the SSO can also inhibit oxidation to some extent. Chen et al. [16] added 10 μm α-tocopherol to the system consisting of SSO and medium chain triacylglycerols (25:75 wt%) and the lag period was 11 days. After adding MAG, the lag period of the system was reduced to 10, 9, and 7 days (0.5, 1.5, 2.5 wt%), respectively, which also indicated that MAG in bulk lipid can reduce the antioxidant capacity of α-tocopherol [37]. As the effect of 2D-MAG on the SSO with/without antioxidants was significantly different, 2D-MAG and α-tocopherol had no effect on the inhibition and synergistic enhancement of SSO oxidation. Conversely, the addition of 2D-MAG reduced the inhibitory effect of α-tocopherol on SSO oxidation. Lee et al. [38] showed that although the addition of quercetin or rutin had an antioxidant effect in stripped oil, they promoted oxidation in non-stripped soybean oil. In this study, a potential possibility was that 2D-MAG in SSO inhibited oxidation mainly by itself oxidizing to avoid SSO from being oxidized.

### 3.8. Effect of 1,3C-2D-TAG on the Oxidation Stability of SSO with α-Tocopherol

**Figure 8:**
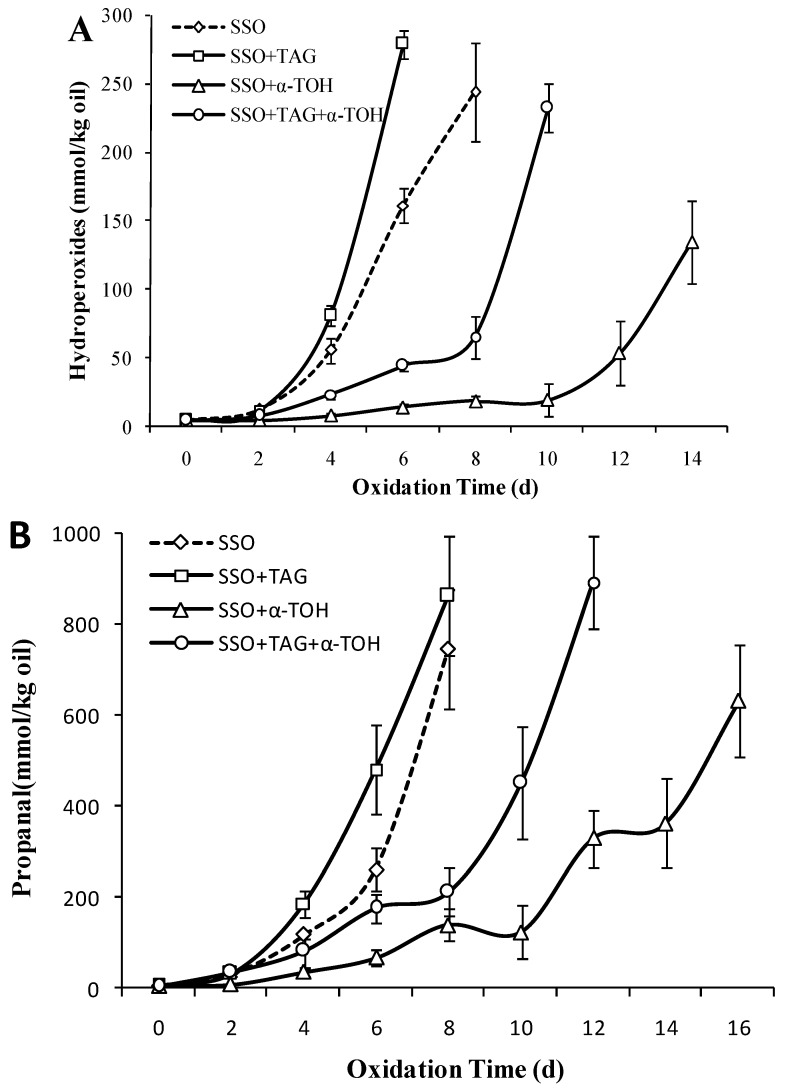
Effect of 1,3C-2D-TAG structured lipids on the content of POV (**A**) and propanal (**B**) of stripped soybean oil with and without α-tocopherol (50 °C).

The oxidative stability of 1,3C-2D-TAG in non/added antioxidant SSO was compared. From Figure 8A, the oxidation induction period of 1,3C-2D-TAG in the SSO system without antioxidant was only 2 days, and then the POV content reached 279.33 mmol/kg in the sixth day, which was significantly higher than that without 1,3C-2D-TAG on the fourth day (161.29 mmol/kg; *p* < 0.05). This indicated that 1,3C-2D-TAG accelerated oxidation and reduced the stability of SSO. Koh et al. [33] showed that the oxidative stability of structured lipids prepared using any enzyme catalysis was significantly lower than that of raw oil, which was related to the changed ratio of modified structured lipids to original triglyceride, and even the cis/trans, conjugated and non-conjugated FA systems affected the oxidation process. When 1,3C-2D-TAG was added to SSO system containing 0.2% α-tocopherol, the lipid oxidation induction period was extended to 8 days, and the POV content rapidly increased to 232.46 mmol/kg (*p* < 0.05) on the tenth day. Compared to the SSO without 1,3C-2D-TAG, 1,3C-2D-TAG reduced the inhibitory effect of antioxidants. As 1,3C-2D-TAG is linked to the highly unsaturated FA DHA at the sn-2 acyl position, it only slightly inhibits the oxidation process of 1,3C-2D-TAG.

The 1,3C-2D-TAG started to produce considerable amounts of propanal on the fourth day, after which the amount of propanal increased rapidly (Figure 8B). The amount of propanal produced by 1,3C-2D-TAG was significantly higher than the SSO group (*p* < 0.05). In addition, the oxidation induction period of 1,3C-2D-TAG in SSO with antioxidant was short (4 days), but the propanal content increased rapidly after 8 days. Compared to the antioxidant SSO without 1,3C-2D-TAG, the addition of 1,3C-2D-TAG led to the accumulation of more propanal, which suggested that 1,3C-2D-TAG reduced the anti-oxidation effect of antioxidants in the SSO system with similar changes in POV content.

### 3.9. Effect of 1,2,3C-TAG on the Oxidation Stability of SSO with α-Tocopherol

**Figure 9:**
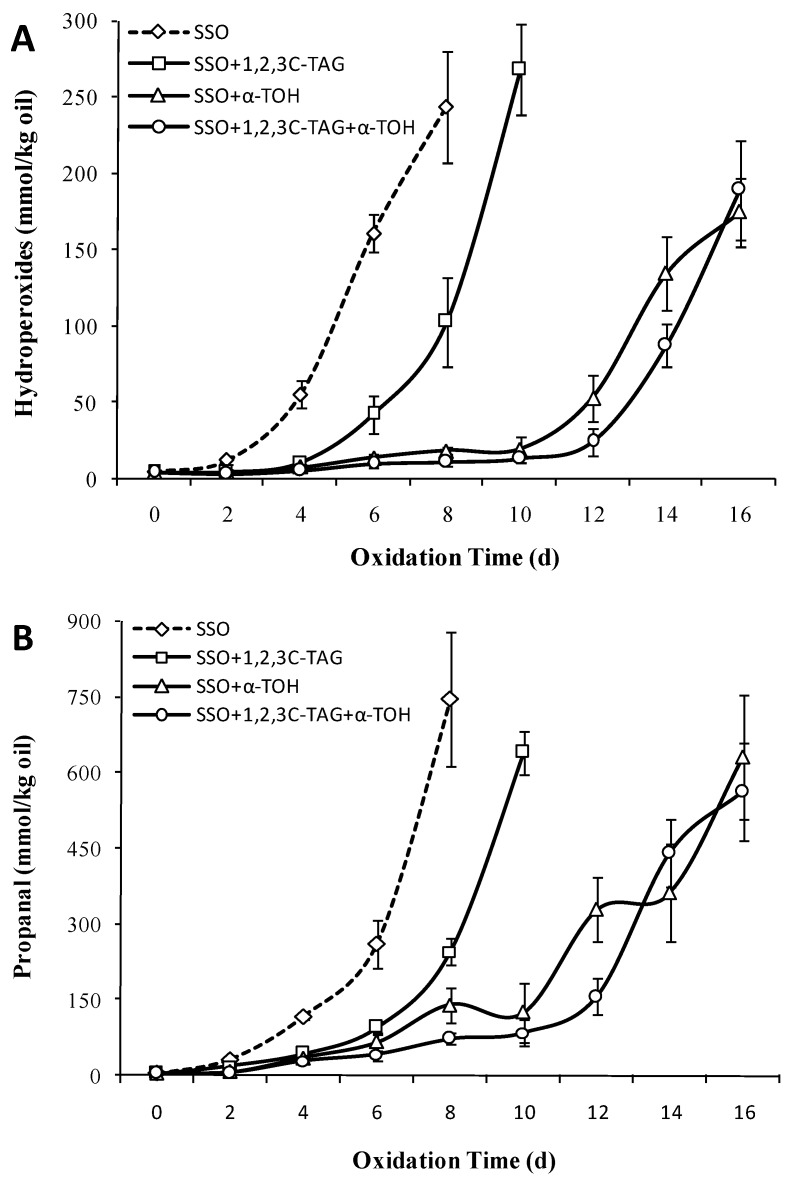
Effect of 1,2,3C-TAG structured lipids on the content of POV (**A**) and propanal (**B**) of stripped soybean oil with and without α-tocopherol (50 °C).

To further study the effect of the sn-2 acyl position and the FAs attached to the sn-2 acyl position on structured lipid oxidation, the oxidative stability of 1,2,3C-TAG was assessed in this study. Figure 9A showed that addition of 5% 1,2,3C-TAG delayed the oxidation lag period of the SSO to 4 days, coupled with a significant increase in POV content. The POV value on the eighth day reached 103.21 mmol/kg, which was significantly lower than that without 1,2,3C-TAG on the eighth day (244.33 mmol/kg (*p* < 0.05)). Compared to the effect of 1,2,3C-TAG on the oxidation of SSO, DHA at 1,3C-2D-TAG sn-2 position induced the oxidation of structured lipids, thereby further promoting the oxidation of SSO. Studies have shown that the consumption of endogenous antioxidants in natural lipid, and the changes in FA composition and FA distribution in triglyceride, which were the main reasons for the changes in structured lipid oxidation stability.

The effect of 1,2,3C-TAG on the content of propanal in the SSO is shown in Figure 9B. Addition of 2% 1,2,3C-TAG significantly affected the content of propanal in the SSO. On the eighth day of storage, the content of propanal in the SSO with 1,2,3C-TAG was 245.45 μmol/kg, which was significantly lower than that of the system without 1,2,3C-TAG (747.12 μmol/kg). Owing to the presence of saturated medium chain FAs linked to the 1,2,3C-TAG glycerol carbon chain, propanal was formed mainly from the oxidation of long-chain monounsaturated FAs in the SSO system [39]. In the presence of antioxidants, 1,2,3C-TAG showed no significant oxidation inhibition effect on SSO. Combined with the effect of 1,2,3C-TAG on the content of POV and propanal in the SSO with antioxidant, we concluded that 1,2,3C-TAG has obvious inhibitory effects on the oxidation of the pure SSO system, although this inhibitory effect was not significant in the presence of antioxidants. Addition of antioxidants improves the antioxidant stability of structured lipids; however, whether different antioxidants differentially affect structured lipids remains to be further studied.

### 3.10. Changes in the DHA Content in Different SSO during Accelerated Oxidation Period

To investigate the relationship between the DHA content at sn-2 position and the oxidation of SSO supplemented with structured lipid, the change in the DHA content at sn-2 position in 2D-MAG and 1,3C-2D-TAG during lipid oxidation was compared. Table 2 shows that there was no DHA either in the free FA or at the sn-2 site of the triglyceride, which provided a blank control basis for the structured lipid to study the DHA content change in the SSO system. After the addition of 20% 2D-MAG to the SSO, the total amount of DHA in the system and the DHA content at the sn-2 site of the structured lipid were 10.16% and 8.69% of FA content, respectively. With continuous oxidation, the DHA content at the sn-2 site in SSO and 2D-MAG began to decline. Among them, the DHA content at sn-2 site decreased to 2.97% on the second day, which was 59.9% lesser than that of the previous day (*p* < 0.05). After the fourth day of oxidation, no DHA was detected at the sn-2 site in 2D-MAG, indicating that the DHA at the sn-2 site was oxidized and its content decreased rapidly. Compared to the changes in the DHA content at the sn-2 site in 2D-MAG, the total DHA content in SSO decreased from 10.16% (Day 0) to 5.22% (Day 4), which indicated that during the oxidation process of 0–3 days, the DHA content at the sn-2 site in 2D-MAG decreased more rapidly than that in the SSO. One possible reason was that monoglycerides at the sn-2 site undergo a transesterification reaction during oxidation, and some sn-2 MAGs are converted to sn-1/sn-3 MAG. Laszlo et al. [29] reasoned that sn-2MAG had a higher proportion of transesterifications due to the position of hydroxyl groups. In addition, the rate of acyl migration in MAG was affected by temperature, solvent, acid, and base, and the length, unsaturation, and distribution direction of acyl group also affected the balance and distribution of MAGs in lipids. When antioxidants were added, the total amount of DHA in the SSO system and the DHA content at the sn-2 position decreased (*p* > 0.05). On the fourth day of oxidation, there was 5.22% total DHA and 3.28% sn-2 DHA in SSO, which was significantly higher than that of the experimental group without antioxidant. Therefore, the antioxidant is able to inhibit not only the oxidation of DHA in the SSO, but also the transesterification of sn-2 DHA to sn-1/sn-3 DHA in the structured lipid.

Compared to that in the 2D-MAG group, the decrease in the DHA content at the sn-2 site in the 1,3C-2D-TAG group was more obvious. On the fourth day of oxidation, no DHA was detected at the sn-2 site in the SSO group and 1,3C-2D-TAG group, suggesting that the oxidation rate of DHA at the sn-2 site in 1,3C-2D-TAG was significantly faster than that in 2D-MAG (*p* < 0.05). Owing to the presence of antioxidants, the oxidation of DHA at the sn-2 site in SSO and 1,3C-2D-TAG was inhibited by both antioxidants, although the inhibitory effect was clearly not as good as that in 2D-MAG. The possible reason is that all the three acyl sites of 1,3C-2D-TAG are occupied with FAs, and there is no transesterification of DHA at the sn-2 site; hence, antioxidants affecting the oxidation of total DHA did not show any obvious effect on the oxidation of DHA at sn-2 site. As caprylic triglyceride did not contain DHA, no DHA content was detected in the 1,2,3C-TAG group.

## 4. Conclusions

To study the oxidative stability and oxidation mechanism of various DHA-containing structured lipids, the content of hydroperoxides and propanal of different structured lipids in the stripped soybean oil system were studied. Results have shown that a small amount (2%) of 2D-MAG and 1,3C-DAG can prolong the oxidation induction period of SSO to some extent. 2D-MAG (2 wt%) inhibited the oxidation of the pure SSO, while addition of 2D-MAG to SSO with α-tocopherol suppressed the inhibitory effect of α-tocopherol on the oxidation of SSO. Inhibition of oxidation was exhausted with the consumption of 2D-MAG, and considerable hydroperoxides was produced. Thus, 2D-MAG promoted oxidation in the SSO.

Similarly, compared to 1,2,3C-TAG, the structured lipids with DHA at the sn-2 acyl site (1,3C-2D-TAG) induced oxidation of structured lipids, thus further promoting the oxidation of SSO. This indicated that the type of acyl FA at sn-2 site considerably affects the degree and speed of oxidation of lipids. Under accelerated oxidation, an antioxidant is required for maintaining the stability of 1,3C-2D-TAG in the SSO and commercial lipid.

## Figures and Tables

**Table 1:** Hydrogen peroxide and propanal content in oil samples before and after purification (25 °C).

Oil types	Hydroperoxides (mmol/kg oil)	Propanal (μmol/kg oil)
Non-stripped oil	16.06 ± 2.43 ^a^	51.31 ± 3.81 ^a^
Stripped oil	2.22 ± 0.32 ^b^	9.83 ± 0.74 ^b^

Different superscripts lowercase letters in the same column indicate significant differences (*p* ˂ 0.05).

**Table 2:** Changes of total and sn-2 DHA content of structured lipids in SSO during storage (50 °C).

Oil	0 day		1 day		2 days		3 days		4 days		5 days	
	Tota DHA	sn-2	Total DHA	sn-2	Total DHA	sn-2	Total DHA	sn-2	Total DHA	sn-2	Total DHA	sn-2
SSO	NF	NF	NF	NF	NF	NF	NF	NF	NF	NF	NF	NF
SSO+2D-MAG	10.16 ± 1.57 ^a^	8.69 ± 1.82 ^a^	8.97 ± 1.13 ^b^	7.42 ± 1.35 ^a^	6.72 ± 0.52 ^c^	2.97 ± 0.55 ^b^	5.25 ± 1.03 ^c^	2.58 ± 0.93 ^b^	3.77 ± 0.82 ^d^	1.37 ± 0.45 ^c^	0.82 ± 0.35 ^e^	NF
SSO+2D-MAG+0.2 α-TOH	10.16 ± 1.57 ^a^	8.69 ± 1.82 ^a^	8.71 ± 1.48 ^b^	8.94 ± 1.66 ^a^	8.01 ± 0.93 ^b^	6.58 ± 0.81 ^b^	6.48 ± 1.17 ^c^	4.62 ± 1.48 ^c^	5.22 ± 1.53 ^c^	3.28 ± 1.26 ^c^	1.63 ± 0.62 ^d^	0.58 ± 0.37 ^d^
SSO+1,3C-2D-TAG	9.03 ± 1.44 ^a^	7.42 ± 0.97 ^a^	8.37 ± 2.26 ^a^	5.02 ± 1.35 ^b^	4.85 ± 1.24 ^b^	1.37 ± 0.48 ^c^	2.03 ± 0.85 ^c^	1.02 ± 0.09 ^d^	NF	NF	NF	NF
SSO+1,3C-2D-TAG+0.2 α-TOH	9.03 ± 1.44 ^a^	7.42 ± 0.97 ^a^	8.69 ± 1.38 ^a^	5.84 ± 1.12 ^b^	6.60 ± 2.04 ^b^	4.43 ± 1.25 ^b^	4.72 ± 1.50 ^c^	2.74 ± 0.89 ^c^	2.85 ± 1.16 ^d^	1.28 ± 0.75 ^d^	NF	NF
SSO+1,2,3C-TAG	NF	NF	NF	NF	NF	NF	NF	NF	NF	NF	NF	NF

“NF” represents “Not Found”; “SSO” represents stripped soybean oil; “SSO+2D-MAG” represents tripped soybean oil with monoglycerides (DHA at sn-2 position); “SSO+2D-MAG+0.2α-TOH” represents stripped soybean oil with monoglycerides (DHA at sn-2 position) and α-tocopherol; “SSO+1,3C-2D-TAG” represents stripped soybean oil with triglyceride (caprylic acid at sn-1,3 and DHA at sn-2 position); “SSO+1,3C-2D-TAG+0.2α-TOH” represents stripped soybean oil with triglyceride (caprylic acid at sn-1,3 and DHA at sn-2 position) and α-tocopherol; “SSO+1,2,3C-TAG” represents stripped soybean oil with caprylic triglyceride. Different superscripts lowercase letters in the same row indicate significant differences (*p* ˂ 0.05).
